# Anti‐GPVI nanobody blocks collagen‐ and atherosclerotic plaque–induced GPVI clustering, signaling, and thrombus formation

**DOI:** 10.1111/jth.15836

**Published:** 2022-08-12

**Authors:** Natalie J. Jooss, Christopher W. Smith, Alexandre Slater, Samantha J. Montague, Ying Di, Christopher O'Shea, Mark R. Thomas, Yvonne M. C. Henskens, Johan W. M. Heemskerk, Steve P. Watson, Natalie S. Poulter

**Affiliations:** ^1^ Institute of Cardiovascular Sciences, College of Medical and Dental Sciences University of Birmingham Birmingham UK; ^2^ Department of Biochemistry, Cardiovascular Research Institute Maastricht (CARIM) Maastricht University Maastricht the Netherlands; ^3^ Department of Cardiology University Hospitals Birmingham Birmingham UK; ^4^ Central Diagnostic Laboratory Maastricht University Medical Centre Maastricht the Netherlands; ^5^ Synapse Research Institute Maastricht Maastricht the Netherlands; ^6^ Centre of Membrane Proteins and Receptors (COMPARE) Universities of Birmingham and Nottingham Midlands UK

**Keywords:** atherosclerotic plaque, glycoprotein VI, nanobody, platelet activation, receptor clustering

## Abstract

**Background:**

The collagen receptor glycoprotein VI (GPVI) is an attractive antiplatelet target due to its critical role in thrombosis but minor involvement in hemostasis.

**Objective:**

To investigate GPVI receptor involvement in platelet activation by collagen‐I and atherosclerotic plaque using novel blocking and non‐blocking anti‐GPVI nanobodies (Nbs).

**Methods:**

Nb effects on GPVI‐mediated signaling and function were assessed by western blot and whole blood thrombus formation under flow. GPVI clustering was visualized in thrombi using fluorescently labeled Nb28.

**Results:**

Under arterial shear, inhibitory Nb2 blocks thrombus formation and platelet activation on collagen and plaque, but only reduces adhesion on plaque. In contrast, adhesion on collagen, but not plaque, is decreased by blocking integrin α2β1. Adhesion on plaque is maintained despite inhibition of integrins αvβ3, α5β1, α6β1, and αIIbβ3. Only combined αIIbβ3 and α2β1 blockade inhibits adhesion and thrombus formation to the same extent as Nb2 alone. Nb2 prevents GPVI signaling, with loss of Syk, Lat, and PLCɣ2 phosphorylation, especially to plaque stimulation. Non‐blocking fluorescently labeled Nb28 reveals distinct GPVI distribution patterns on collagen and plaque, with GPVI clustering clearly apparent on collagen fibers and less frequent on plaque. Clustering on collagen fibers is lost in the presence of Nb2.

**Conclusions:**

This work emphasizes the critical difference in GPVI‐mediated platelet activation by plaque and collagen; it highlights the importance of GPVI clustering for downstream signaling and thrombus formation. Labeled Nb28 is a novel tool for providing mechanistic insight into this process and the data suggest Nb2 warrants further investigation as a potential anti‐thrombotic agent.

**Essentials**

• Inhibition of glycoprotein VI (GPVI) is a promising approach to prevent thrombosis while maintaining hemostasis.

• Anti‐GPVI nanobody2 blocks GPVI clustering, signaling, and thrombus formation on collagen.

• Fluorescently labeled anti‐GPVI nanobody28 can be used to visualize GPVI in aggregates.

• Anti‐GPVI nanobodies show potential as novel anti‐thrombotics and GPVI imaging tools.

## 1 INTRODUCTION

Cardiovascular diseases (CVD) associated with thrombosis, including acute coronary syndromes and stroke, are among the world's leading causes of death, with 17.9 million deaths in 2019.^1^ Improved anti‐thrombotic therapies with reduced bleeding side effects are required to increase survival, improve patients' quality of life, and ease the financial burden of CVD. Platelets are small, highly reactive anucleate blood cells, known for their major contribution to hemostasis, but also play a central role in thrombotic events, as well as in a myriad of other (patho)physiological processes.^2^

Platelets become activated by extracellular matrix proteins exposed upon vessel damage, as well as by atherosclerotic plaque components uncovered by plaque rupture or erosion.^3^ The formation and progression of atherosclerotic plaques is caused by an inflammatory process within the vessel wall, mediated by macrophage incorporation, vascular cell proliferation, and lipid deposition. The lipid‐rich necrotic core of an atherosclerotic plaque is sealed with a collagen‐rich cap.^4^ Rupture of the fibrous cap induces platelet activation. Weak platelet agonists present are lysophosphatidic acid,^5^ sphingosine‐1‐phosphate,^6^ fibronectin, and fibrin(ogen).^3^ However, plaque‐mediated platelet activation has been shown to be predominantly induced by collagens.^7^ Intriguingly, these collagens, mostly of types I and III,^3^, ^7^, ^8^, ^9^ have been observed to mediate platelet activation via glycoprotein VI (GPVI) but not integrin α2β1, in contrast to *in vitro* studies on immobilized collagens in which both receptors play a role.^7^, ^8^, ^10^, ^11^

GPVI is a platelet and megakaryocyte‐specific ~62 kDa immunoglobulin‐like transmembrane receptor. It is expressed at the platelet surface in complex with the immunoreceptor tyrosine‐based activation motif (ITAM)‐containing Fc receptor γ‐chain (FcRγ)^12^ and is considered the main signaling receptor in platelets.^13^, ^14^ Its primary ligand is collagen, but it has been shown to act as a multi‐ligand receptor, binding to substrates including fibrin, fibrinogen, or laminin.^15^ Upon ligand binding, the GPVI–FcRγ complex initiates intracellular signaling by phosphorylation of several downstream molecules, including Syk, LAT, and PLCɣ2, inducing calcium mobilization and resulting in platelet activation and thrombus formation.^16^, ^17^ Signaling strength and duration is amplified by clustering of GPVI, induced by platelet attachment to collagen fibers.^18^, ^19^, ^20^, ^21^ The restricted expression of GPVI to platelets and megakaryocytes and its minor role in hemostasis^16^, ^22^, ^23^ make GPVI a target for novel anti‐atherothrombotic therapies.^24^

We have recently described a series of novel anti‐GPVI nanobodies (Nbs), several of which are potent GPVI inhibitors.^25^ Cameloid‐derived Nbs contain only a single chain of the variable region of full‐size antibodies, making them smaller than antibody F(ab) fragments (15 kDa versus 50 kDa), while maintaining high binding affinities and antigen specificity.^26^ Nbs display several beneficial features, such as stability, tissue penetration, and a low immunogenic impact, making them suitable for multiple applications including imaging^27^ and as therapeutic agents.^28^, ^29^

In this study we have compared the activation potential of atherosclerotic plaque homogenate to fibrillar collagen type I. We used plaque homogenate as a physiological ligand to evaluate the effectiveness of the novel anti‐GPVI Nb2 in inhibiting whole blood thrombus formation. In addition, we introduce a fluorescently labeled non‐inhibitory anti‐GPVI Nb (Nb28), allowing for the first time investigation of GPVI localization and clustering in platelets forming thrombi under flow, and assess the effect of GPVI inhibitors on this. We show Nb2 is a potent inhibitor of collagen‐induced platelet activation, which results in a strong reduction of GPVI downstream signaling and subsequent thrombus formation. Further, we propose that this inhibitory mechanism is mediated by complete disruption of GPVI receptor clustering. These findings support the suitability of Nb2 as an anti‐thrombotic agent, especially in atherothrombosis.

## 2 MATERIALS AND METHODS

### 2.1 Antibodies and reagents

Nanobodies raised against the extracellular domain of GPVI were expressed as previously described.^25^ Nb28 was labeled using AlexaFluor 647 NHS ester (Thermo Fisher Scientific). Fibrillar collagen I (Horm) was from Nycomed, P11 from TOCRIS, Eptifibatide from GSK, JBS5 from Santa Cruz, GoH3 from Invitrogen, and 6F1 mAb was a gift from Barry Coller (Rockefeller University).

### 2.2 Histology of human atherosclerotic plaque and generation of pooled homogenates

Ten patients undergoing carotid endarterectomy at the Queen Elizabeth Hospital in Birmingham gave informed consent (ethical approval: North West – Haydock Research Ethics Committee 20/NW/0001) and donated their extracted plaque material, which was snap frozen in liquid nitrogen and stored at −80°C. Before use, the samples were divided in half; one half was used for histological analysis and the other was utilized to generate a pooled plaque homogenate.

Histological staining to assess plaque composition was conducted on OCT (Sakura) embedded, cryo‐sectioned (6 μm) samples, co‐stained for Ca^2+^ and global collagen content with von Kossa (Merck) and Van Gieson (Atom Scientific), respectively. Further sections were investigated for either lipid content with OilRedO (Sigma Aldrich) or with hematoxylin and eosin (Atom Scientific) for tissue orientation. All stains were used in accordance with the respective manufacturer's instructions, before being mounted (DPX, Merck) and imaged at 20× magnification with an Axio Slide scanner (Zeiss).

To generate plaque homogenate, frozen plaque samples were mechanically pulverized with a glass mortar and pestle in liquid nitrogen to generate a fine powder. This was pooled and dissolved in phosphate‐buffered saline (PBS), solubilized by sonification (5 × 10 s), and a short centrifugation step to remove debris. The supernatant was aliquoted and stored at −80°C. Protein concentration was determined using a standard Bradford protein assay and demonstrated to be 14 mg/ml. The plaque homogenate was tested at various concentrations between 0.5 mg/ml up to 5 mg/ml (in line with the literature^10^, ^11^) in whole blood microfluidics and the lowest concentration that gave maximal thrombus formation and platelet activation was used – 500 μg/ml.

### 2.3 Platelet isolation

Blood was collected into 4% sodium citrate, after informed consent, from drug‐free, healthy volunteers in accordance with the Declaration of Helsinki and ethical approval granted by University of Birmingham internal ethical review (ERN‐11‐0175). Washed platelets were isolated as previously described.^30^ Briefly, acid citrate dextrose (ACD, 1:10 v/v) was added to blood then platelet‐rich plasma (PRP) was obtained by centrifugation at 200 × g for 20 min at room temperature (RT). Washed platelets were isolated by centrifugation of the PRP in the presence of 0.2 μg/ml prostacyclin (1000 × g, 10 min, RT), then washed in modified Tyrode's buffer (129 mM NaCl, 0.34 mM Na_2_HPO_4_, 2.9 mM KCl, 12 mM NaHCO_3_, 20 mM HEPES, 5 mM glucose, 1 mM MgCl_2_; pH 7.3) supplemented with ACD, centrifuged again in the presence of 0.2 μg/ml prostacyclin (1000 x g, 10 min, RT), then the platelet pellet was resuspended in modified Tyrode's–HEPES buffer and left to rest for 30 min before being used in experiments.

### 2.4 Platelet spreading

Glass coverslips were coated with 10 μg/ml fibrillar collagen I or 500 μg/ml plaque homogenate (overnight, 4°C) then blocked with 5 mg/ml bovine serum albumin (1 h, RT). Platelets were preincubated with PBS or 500 nM Nb2, spread for 45 min (37°C), fixed, permeabilized, and labeled with phalloidin‐Alexa488. Spreading analysis was as described in Pike et al.^31^

### 2.5 Ca^2+^ mobilization in live spread platelets

Glass bottom dishes (MatTek Corp) were coated and blocked, as above. Washed platelets were loaded with 1 μM Oregon Green‐488 BAPTA‐1‐AM (Thermo Fisher Scientific) and spread for 45 min. Pre‐ and post‐treatment videos were captured and platelet Ca^2+^ mobilization analyzed as detailed in supporting information.

### 2.6 Western blotting

Washed platelets preincubated with PBS, 500 nM Nb2 or Nb53, were stimulated with 10 μg/ml fibrillar collagen I or 500 μg/ml pooled plaque homogenate under stirring conditions as previously described. Whole cell lysates were then probed for total phosphotyrosine (4G10, Millipore), PLCγ2‐pY1217 (Cell Signaling, 3871S), LAT‐pY200 (Abcam, ab68139), Syk‐pY525/526 (Cell Signaling, 2710S), and total Syk (4D10, Santa Cruz).^30^ Bands were visualized using autoradiography film, and Odyssey Fc System (LI‐COR Biosciences) and quantified in Image Studio Lite v5.2.

### 2.7 Light transmission aggregometry

Platelet aggregation was assessed using a light transmission aggregometer (Model 700, ChronoLog; 37°C, 1200 rpm), washed platelets (2 × 10^8^/ml) were stimulated with 10 μg/ml fibrillar collagen I following vehicle, 500 nM Nb2, Nb53, or Nb28 preincubation.

### 2.8 Competition assays

For flow cytometry, washed platelets were preincubated with 500 nM Nb2 or PBS, followed by addition of 100 nM Nb28‐AF647 and immediate fixation (4% paraformaldehyde); 50 000 events were acquired (BD Accuri C6 flow cytometer) and plotted in FlowJo (BD). For ELISA, Nb28 and Nb21 binding to immobilized GPVI‐Fc in the presence of Nb2 was assessed as previously described.^25^

### 2.9 GPVI shedding assay

Washed platelets (2 × 10^8^/ml) were recalcified with calcium chloride (1 mM) before treatment with 500 nM Nb2 or Nb53 for 2 h, under static conditions at RT. Platelets were also stimulated with 5 mM N‐ethylmaleimide (NEM), as a positive control for GPVI shedding. After treatment, platelets were incubated with phycoerythrin‐conjugated anti‐GPVI antibody (HY101, BD Biosciences) for 30 min before dilution in PBS and GPVI surface expression (median fluorescence intensity [MFI]) measured by flow cytometry (10 000 events acquired, BD Accuri C6 flow cytometer, plotted in FlowJo). Reduction in platelet GPVI MFI compared to resting controls represented GPVI shedding.

### 2.10 Whole blood microfluidics

Citrated whole blood was preincubated with vehicle, 500 nM Nb, or 20 μg/ml 6F1, thrombin‐inhibited (40 μM PPACK) and recalcified (3.75 mM MgCl_2_ and 7.5 mM CaCl_2_) then perfused over plaque and collagen microspots in a Maastricht flow chamber at 1000/s, as described.^32^ Platelets were labeled for activation markers: AF568‐annexin A5 (for phosphatidylserine exposure, procoagulant platelets, Thermo Fisher), AF647 anti‐CD62P mAb (for CD62P expression, α‐granule secretion, BioLegend), and anti‐fibrinogen fluorescein isothiocyanate Ab (for fibrinogen binding, integrin αIIbβ3 activation, Dako) and endpoint images obtained on an EVOS AMF4300 microscope (Life Technologies). Thrombus formation and platelet activation was quantified. See supporting information for full details of image capture and analysis.

### 2.11 Visualization of GPVI clustering

Flow adhesion was performed as above, with GPVI visualized by addition of 100 nM Nb28‐AF647 prior to flow.

### 2.12 Statistical analysis

Data is shown as mean ± standard deviation (SD). Statistical tests are indicated in figure legends and were performed in GraphPad Prism v7.

## 3 RESULTS

### 3.1 Atherosclerotic plaque homogenate activates platelets under static and flow conditions

Human atherosclerotic plaque has been previously described as a heterogeneous substrate, rich in collagen type I and III.^3^, ^7^, ^11^ Histology on sections of the plaques used in this study highlighted their collagen‐rich nature but demonstrated differences in composition and structure of individual plaques (Figure S1 in supporting information). Hence, we pooled 10 atherosclerotic plaques and assessed the platelet activatory potential of the pooled homogenate compared to standard fibrillary collagen I in platelet activation assays.

**FIGURE 1:**
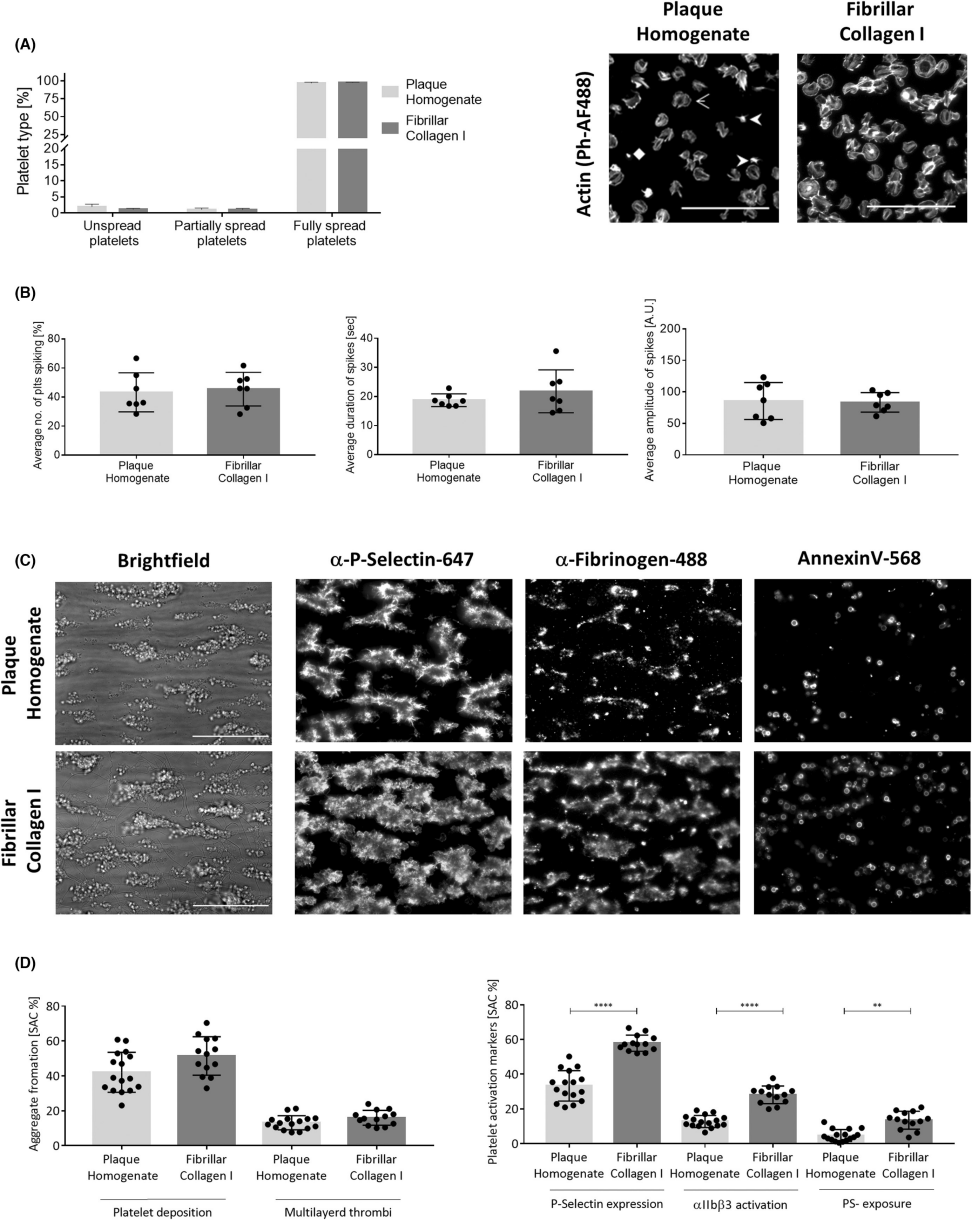
Plaque homogenate supports platelet activation under static and flow conditions. (A) Platelets were spread on fibrillar collagen I (10 μg/ml) and plaque homogenate (500 μg/ml) and percentage of unspread, partially spread, or fully spread platelets quantified. Spreading states are indicated on the plaque representative image with diamond (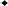 ), unspread; stealth (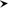 ), partially spread; or open arrow (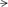 ), fully spread. Six fields of view containing a total of 900–1200 platelets per condition per experiment were analyzed; *n* = 3. (B) Ca^2+^ spiking of spreading platelets loaded with 1 μM BAPTA‐Oregon green Ca^2+^ indicator dye. Percentage of platelets exhibiting spikes, spike duration, and amplitude were assessed (*n* = 7, unpaired *t*‐test). (C) Representative images of aggregate formation and platelet activation on fibrillar collagen I and plaque homogenate microspots in the Maastricht flow chamber perfused with thrombin‐inhibited whole blood at 1000/s. (D) Quantitation of aggregate formation and platelet activation (*n* = 14–16, one‐way analysis of variance). *p < 0.05, **p < 0.005, ***p <0 .0005, ****p < 0.0001, scale bars = 50 μM. PS, phosphatidylserine; SAC, surface area coverage; graph are mean ± standard deviation.

Plaque homogenate supported platelet spreading to a similar extent as fibrillar collagen I (Figure 1A). Next, we investigated Ca^2+^ mobilization, as it represents the latter stages of the GPVI signaling pathway. We performed live‐cell fluorescence imaging of Ca^2+^ mobilization in individual platelets on the two substrates using the Ca^2+^ indicator dye Oregon green 488 BAPTA‐1‐AM (Video S1 in supporting information). No significant difference in percentage of Ca^2+^ spiking platelets, or spike duration or amplitude between plaque and collagen I was observed (Figure 1B). The ability of the plaque homogenate to stimulate platelet adhesion, activation, and thrombus formation under flow at arterial shear (1000/s) was also assessed in comparison to collagen I (Figure 1C,D). Platelet adhesion (deposition) and thrombus size (multilayer) on plaque was not significantly different from collagen I. Plaque induced platelet α‐granule release (P‐selectin), integrin αIIbβ3 activation (fibrinogen binding), and phosphatidylserine (PS) exposure (annexin V; Figure 1C,D). However, collagen I induced significantly more (~50%) platelet activation in these three measured parameters (Figure 1C,D). Taken together, these results indicate that plaque homogenate supports platelet spreading, Ca^2+^ mobilization, and thrombus formation. However, collagen I is a more potent ligand, stimulating greater platelet activation during thrombus formation than plaque. Nevertheless, plaque is a more physiological ligand and can therefore be used to assess the effect of our recently generated anti‐GPVI Nb2^25^ in platelet activation.

### 3.2 Platelet activation by plaque homogenate under flow is integrin independent

**FIGURE 2:**
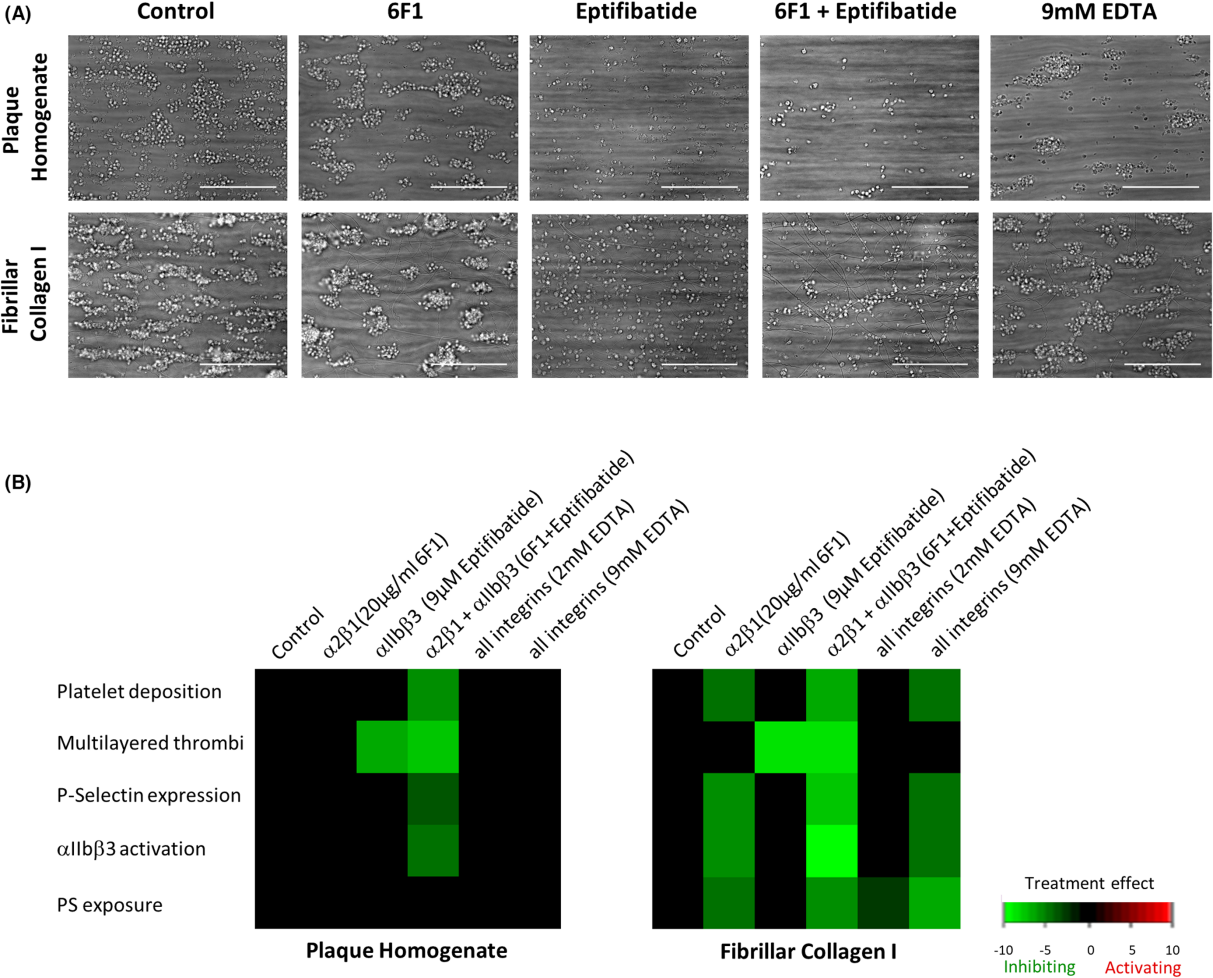
Platelet activation induced by plaque homogenate is integrin independent. (A) Representative brightfield images of thrombi formed on plaque homogenate and fibrillar collagen I in the presence of vehicle (PBS) or integrin inhibitors. Scale bars = 50 μM. (B) Heatmaps summarizing significant effects of the indicated treatments. For each parameter, raw data across all surfaces and donors was univariately scaled (0–10), and control values then subtracted from treatment values, with only significant (*p* < 0.05) changes displayed. Green indicates significant reduction. *n* = 3–5, one‐way analysis of variance. EDTA, ethylenediaminetetraacetic acid; PBS, phosphate‐buffered saline; PS, phosphatidylserine. For raw data see Figure S5 in supporting information.

Platelet activation by plaque has previously been shown to be primarily mediated through GPVI, and independent of the collagen binding integrin α2β1.^7^, ^8^, ^10^, ^11^ However, platelets express multiple integrin receptors and plaque contains several integrin substrates. Therefore, we investigated the potential involvement of other platelet integrins in thrombus formation under arterial shear using blocking reagents to α5β1 (fibronectin; JBS5), α6β1 (laminin; G0H3), αvβ3 (vitronectin; P11), αIIbβ3 (fibrinogen; eptifibatide), and α2β1 (collagen; 6F1). Representative images and subtraction heatmaps, showing only significant differences from controls, are shown in Figure 2. Our results confirmed inhibiting α2β1 had no effect on thrombus formation on plaque, however it reduced platelet deposition and activation markers on collagen I (Figure 2B; Figure S2 in supporting information). Integrin αIIbβ3 inhibition blocked thrombus propagation (multilayer) on both substrates, as expected (Figure 2A,B). However, other platelet activation parameters (P‐selectin expression, integrin activation, PS exposure) were not affected on either substrate (Figure 2B; Figure S2). Inhibition of the other individual integrins did not affect thrombus formation parameters on either substrate (Figure S3 in supporting information). Combined blockade of integrins α2β1 and αIIbβ3 had a greater effect than blocking either alone, and resulted in significant inhibition of platelet adhesion, thrombus formation, and platelet activation on both plaque and collagen I (Figure 2, Figure S2). To investigate this further, we inhibited all integrins by removing free ions with ethylenediaminetetraacetic acid (EDTA). Thrombus formation on plaque and collagen I was not affected by 2 mM EDTA;^33^, ^34^ however, at higher concentrations (9 mM)^35^ a significant decrease in thrombus formation and platelet activation markers was observed on collagen I. On plaque a slight, but not significant, reduction was noted with 9 mM EDTA (Figure 2A, Figure S2). These results indicate that plaque‐induced platelet adhesion and activation is largely independent of individual integrin activation.

### 3.3 Nb2 effectively blocks atherosclerotic plaque‐induced platelet activation and thrombus formation

**FIGURE 3:**
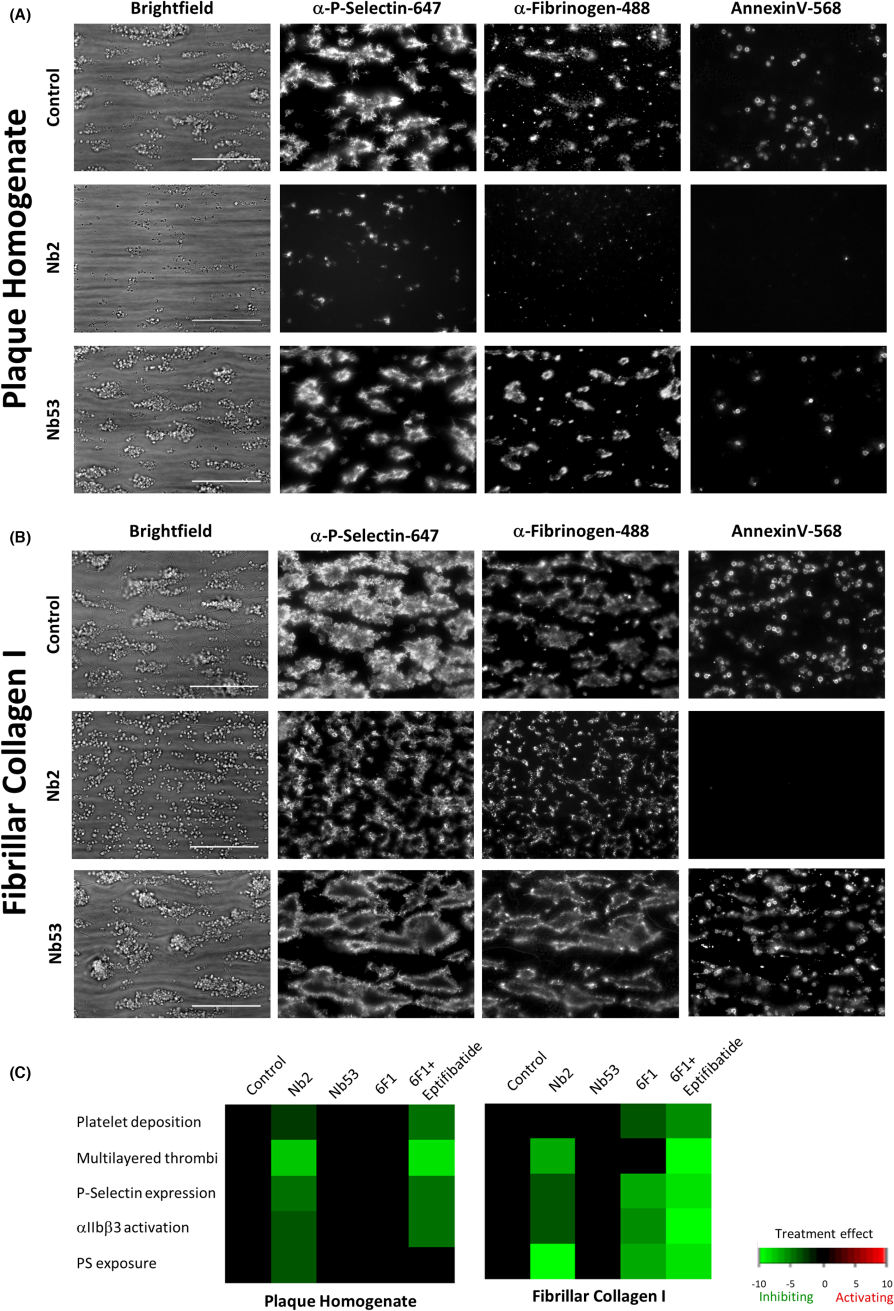
Anti‐GPVI Nb 2 inhibits platelet activation at arterial shear rates. Blood was preincubated with either vehicle (PBS), 500 nM Nb2, or negative control Nb53 for 10 min and then perfused at 1000/s over (A) plaque homogenate or (B) fibrillar collagen I. Brightfield images give information about thrombus size and morphology, while α‐P‐selectin is used to assess α‐granule secretion, α‐fibrinogen to indicate αIIbβ3 activation, and Annexin V to approximate procoagulant activity by PS exposure. Scale bars = 50 μM. (C) Heatmaps, generated by subtracting the univariantly scaled values of the vehicle from the scaled values of the respective treatment for each parameter with only significant (*p* < 0.05) changes indicated. Green indicates significant reduction. *n* = 5–8, one‐way analysis of variance. GPVI, glycoprotein VI; PBS, phosphate‐buffered saline; PS, phosphatidylserine. For raw data see Figure S5 in supporting information.

Of the 54 novel anti‐GPVI Nbs we recently generated,^25^ three have been used in this study: Nb2, a potent inhibitor of GPVI–collagen interactions; Nb53, a weak GPVI binder that is non‐inhibitory and was used as an isotype, negative, control in all experiments; Nb28, a non‐inhibitory Nb that strongly binds GPVI and has been used to label and visualize GPVI for microscopy. The non‐inhibitory nature of Nb53 and Nb28 was confirmed by platelet aggregation in response to collagen (Figure S4 in supporting information). To further characterize the potential anti‐thrombotic properties of Nb2 we tested its effect on plaque‐mediated platelet activation under flow. Blood was pre‐treated with 500 nM Nb, a concentration previously shown to completely block collagen‐induced platelet aggregation,^25^ before being perfused over plaque (Figure 3A) and collagen I (Figure 3B) microspots at arterial shear (1000/s) and thrombus formation and platelet activation was assessed. Significant effects on thrombus formation and platelet activation were summarized into subtraction heatmaps, where bright green indicates strong, statistically significant, inhibition (Figure 3C, Figure S5 in supporting information). On plaque, Nb2 inhibited platelet adhesion and thrombus formation, as well as reduced platelet α‐granule secretion, integrin αIIbβ3 activation, and PS exposure. On collagen I, Nb2 reduced thrombus formation and platelet activation markers, but did not affect platelet adhesion. On both agonists, the negative control Nb53 had no effect. These results demonstrate that Nb2 is a potent inhibitor of platelet activation by both plaque and collagen I, but plaque exhibits more selective dependency on GPVI for thrombus formation.

### 3.4 Nb2 strongly inhibits collagen‐ and atherosclerotic plaque–induced GPVI signaling

**FIGURE 4:**
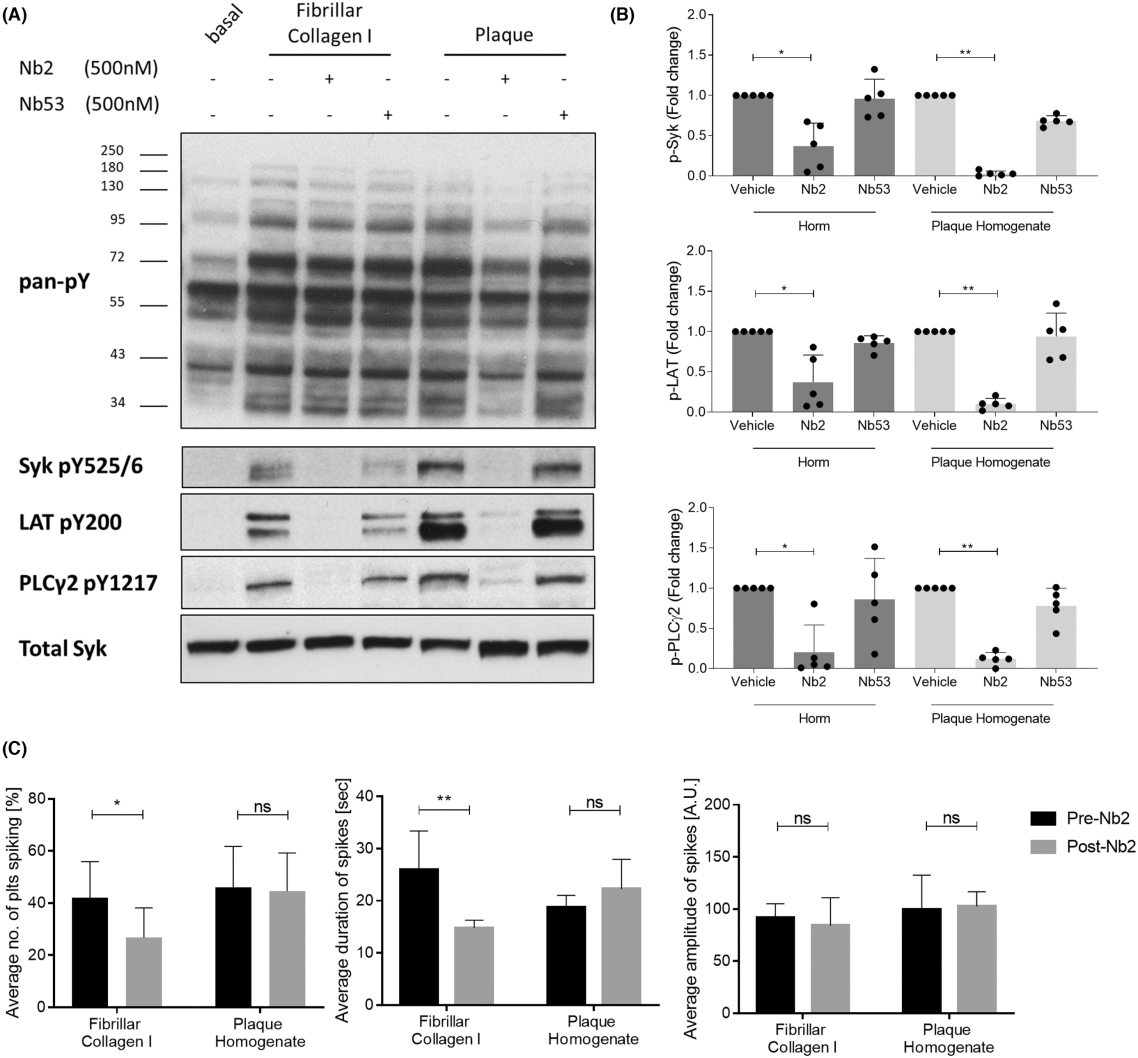
Nb2 strongly inhibits collagen‐ and atherosclerotic plaque‐induced glycoprotein VI (GPVI) signaling. (A) Representative western blots and (B) quantification of phosphorylation of GPVI downstream signaling proteins following stimulation of washed platelets with fibrillar collagen I or plaque for 180 s in the presence or absence of Nb2 or negative control Nb53 (*n* = 5, one‐way analysis of variance [ANOVA]). (C) Ca^2+^ spiking of spreading platelets loaded with 1 μM BAPTA‐Oregon green Ca^2+^ indicator dye were assessed for percentage of platelets exhibiting spikes, as well as spike duration and amplitude, before and after addition of 500 nM Nb2 or Nb53 (*n* = 4, two‐way ANOVA ). Graphs are mean ± standard deviation, ns, not significant, *p < 0.05, **p < 0.005.

To investigate the effect of Nb2 on GPVI signaling we used western blot to examine downstream phosphorylation following stimulation with plaque or collagen I. Both substrates induced strong tyrosine phosphorylation in platelets (Figure 4A). Nb2 addition caused a visible reduction in platelet global tyrosine phosphorylation to plaque stimulation, and strongly inhibited phosphorylation of GPVI downstream signaling proteins Syk Y525/526, LAT Y200, and PLCɣ2 Y1217 in both plaque and collagen I stimulated platelets (Figure 4A). Nb53 had no effect. Quantitation (Figure 4B) revealed strong and consistent reduction of phosphorylation by Nb2 in response to plaque, whereas a slightly weaker and more variable response was observed to collagen I, probably reflecting the involvement of other platelet receptors.^36^

To assess Ca^2+^ signaling, washed platelets loaded with the Ca^2+^ indicator dye were preincubated with Nb2 and spread on collagen I or plaque. However, no platelets adhered under these conditions. We therefore spread platelets onto collagen I or plaque before adding Nb2. Ca^2+^ mobilization was quantified both pre‐ and post‐Nb2 addition (Figure 4C). Nb2 significantly reduced the percentage of Ca^2+^ spiking platelets on collagen I, but had no effect on those spreading on plaque. Nb2 had no effect on spike duration or amplitude on either substrate. The negative control Nb53 had no effect on any parameter (data not shown). Taken together these results show that Nb2 effectively inhibited GPVI‐mediated signaling in response to both collagen I and plaque, but was unable to reverse plaque‐mediated Ca^2+^ signaling in adhered platelets.

### 3.5 Non‐inhibitory Nb28 and inhibitory Nb2 have distinct binding sites on GPVI

**FIGURE 5:**
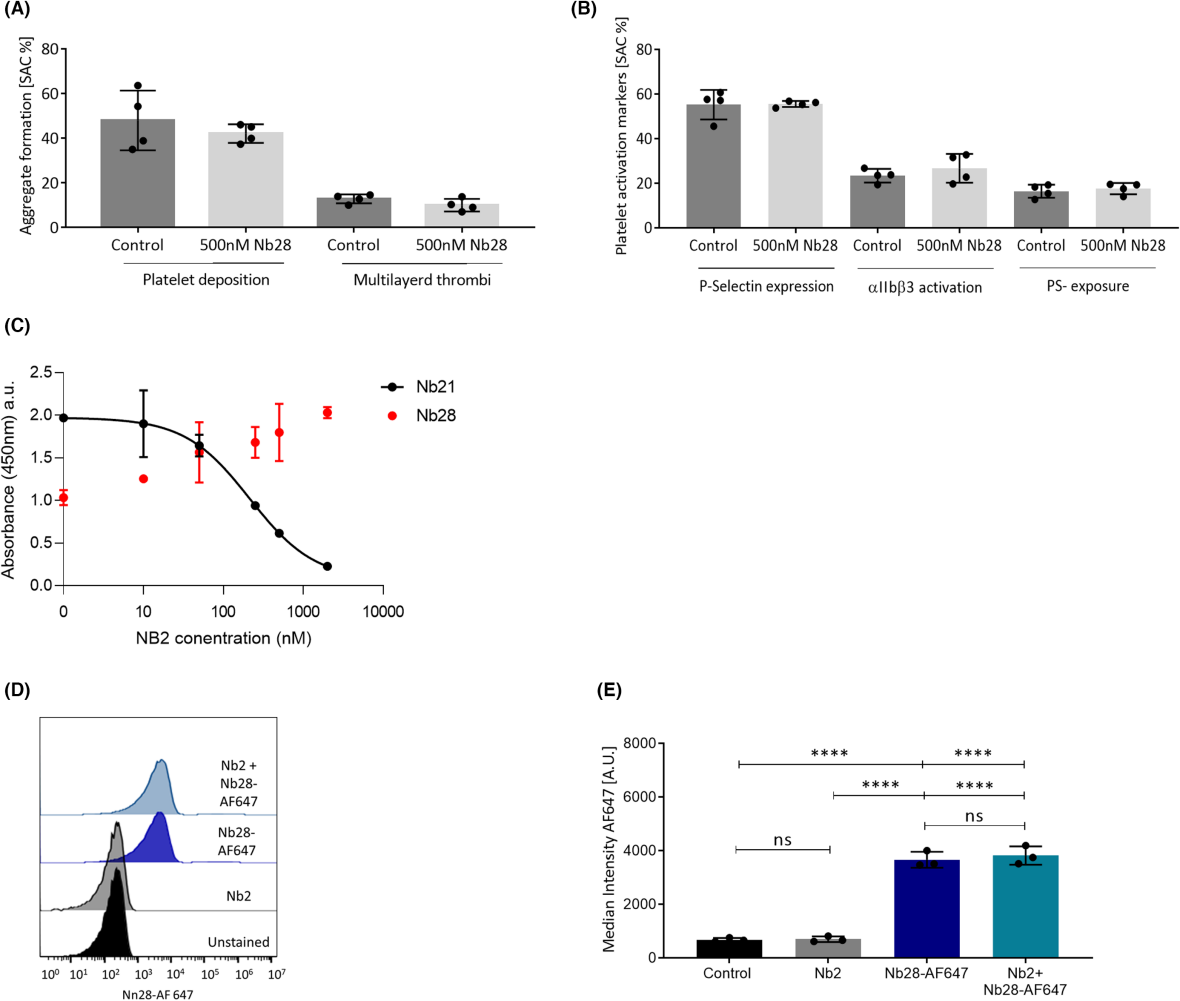
Nb28 does not affect adhesion and activation under flow or compete with Nb2 binding. Thrombin‐inhibited whole blood was preincubated with either vehicle (PBS) or 500 nM of unlabelled Nb28 and then flowed over fibrillar collagen I utilizing the Maastricht flow chamber at 1000/s and assessed for (A) platelet deposition and multilayer thrombi formation and (B) platelet activation markers: PS‐exposure, P‐selectin expression, and integrin αIIbβ3 activation. Each data point represents one donor (*n* = 4), one‐way analysis of variance (ANOVA) assessed significance. (C) A solid‐phase binding assay measuring Nb28 and Nb21 binding to recombinant GPVI in the presence of increasing concentrations of Nb2. Competition between Nb2 and labeled Nb28‐AF647 binding on platelets was assessed by flow cytometry. Washed platelets were preincubated with 500 nM Nb2 or vehicle (PBS) for 10 min, then labeled with 100 nM Nb28‐AF647 and 50 000 events acquired. (D) Representative histograms and (E) quantitative analysis of median fluorescence intensity (*n* = 3). Graph is mean ± standard deviation, two‐way ANOVA; ns, not significant, ****p < 0.0001. GPVI, glycoprotein VI; PBS, phosphate‐buffered saline; PS, phosphatidylserine; SAC, surface area coverage.

To visualize the effect of Nb2 on GPVI localization we required a non‐inhibitory anti‐GPVI Nb that could be used in imaging studies. Nb28 strongly binds GPVI but does not inhibit GPVI–collagen interactions^25^ (Figure S4). To further test the validity of using Nb28 in imaging studies we confirmed that it did not inhibit thrombus formation (Figure 5A) and subsequent expression of platelet activation markers on collagen I under flow (Figure 5B). To verify that Nb28 binds a distinct site and does not interfere with Nb2 GPVI binding a competition ELISA was used (Figure 5C). Nb21, another inhibitory Nb known to bind to a similar epitope in GPVI as Nb2, was used as a control. Nb21 signal decreased due to competition with Nb2 whereas Nb28 signal did not change, suggesting a distinct binding site from Nb2. We fluorescently labeled Nb28 with AlexaFluor647 (Nb28‐AF647) and assessed binding to platelets, as well as competitive binding with inhibitory Nb2, using flow cytometry (Figure 5D). Quantification of flow cytometry experiments revealed no significant difference between platelets labeled with Nb28‐AF647 alone or those pre‐incubated with unlabeled Nb2 (Figure 5E), confirming distinct epitopes on GPVI and making Nb28‐AF647 suitable for imaging of GPVI in experiments where Nb2 is also present.

### 3.6 Nb2 disrupts GPVI clustering

**FIGURE 6:**
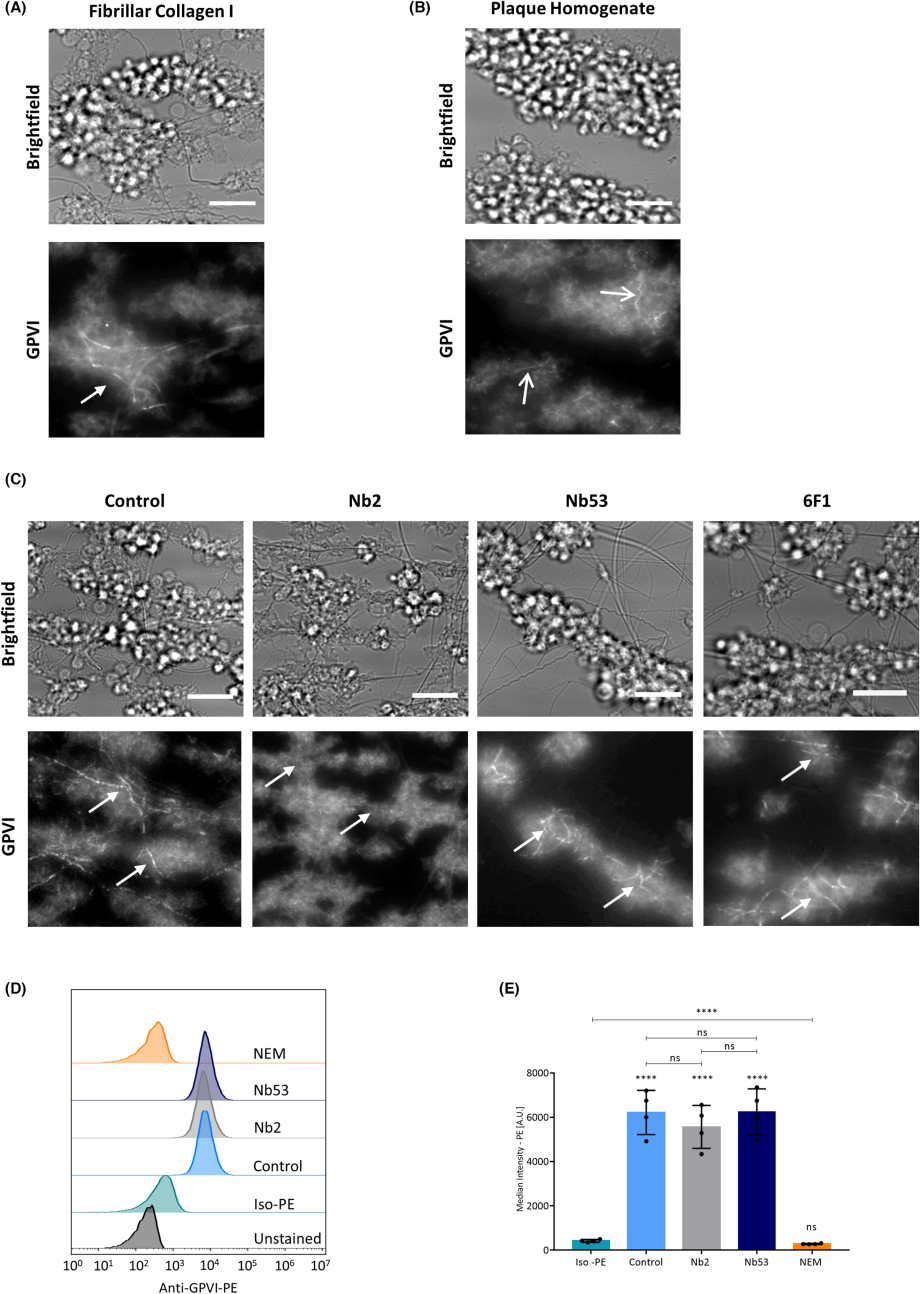
Anti‐GPVI Nb2 disrupts GPVI clustering along collagen fibers in flow. (A) Clustering of Nb28‐AF647 labeled GPVI along collagen I fibers (arrows) in whole blood perfused over collagen I (1000/s). Collagen fibers are visible in the brightfield image. (B) In flow over plaque, some fibrillar clusters of GPVI were seen (open arrows) but GPVI was mostly diffuse. (C) Preincubation of platelets with 500 nM Nb2 disrupted the clustering on collagen I; negative control Nb53 or inhibition of α2β1 with 5 μg/ml 6F1 did not affect clustering of GPVI. Arrows indicate position of collagen fibers seen in brightfield images, *n* = 5–7, scale bars = 10 μM. Washed platelets (2 × 10^8^/ml) were incubated with 500 nM Nb2, Nb53, or 5 mM NEM (stimulator of GPVI shedding) for 2 h before flow cytometry measurement of anti‐GPVI (HY101)–PE binding. (D) Representative histogram of MFI of anti‐GPVI–PE. (E) MFI of platelets with isotype‐PE antibody or anti‐GPVI‐PE labeled control platelets, Nb2‐ and Nb53‐treated platelets, and NEM‐treated platelets. Mean ± standard deviation, *n* = 4, one‐way analysis of variance; ns, not significant, ****p < 0.0001. Significance is shown compared to isotype control or between the treatments denoted by the bars. GPVI, glycoprotein VI; MFI, median fluorescence intensity; NEM, N‐ethylmaleimide; PBS, phosphate‐buffered saline; PE, phycoerythrin; PS, phosphatidylserine.

We have previously shown that GPVI clustering contributes to sustained GPVI signaling in spread platelets on collagen I.^19^ To see if GPVI clustering occurs in platelets forming thrombi under flow we pre‐incubated blood with Nb28‐AF647, before perfusing it over collagen I and taking fluorescent images. GPVI was clearly seen to be enriched at visible collagen fibers, forming long bright clusters in all thrombi (Figure 6A). As platelet activation by plaque was also highly GPVI dependent we investigated GPVI distribution on platelets adhering and forming thrombi on plaque. No collagen fibers were visible in the brightfield image of the plaque; however, in some areas GPVI was seen to form brighter clusters that ran across several platelets in a thrombus, reminiscent of those seen in collagen I (Figure 6B). To investigate the effect of Nb2 on GPVI localization we preincubated blood with Nb2 and Nb28‐AF647 before flow. As very few platelets adhered to plaque in the presence of Nb2 we only investigated GPVI distribution in platelets perfused over collagen I, where clustering was also easily visualized (Figure 6C). Under control conditions, GPVI clustered along the collagen fibers. However, pre‐treatment with Nb2 disrupted the GPVI localization and no organized clustering along collagen fibers was seen. Control Nb53 and 6F1 (integrin α2β1 inhibitor) had no effect on GPVI localization or clustering in the platelets that adhered and formed thrombi on collagen I. GPVI is known to be shed from the platelet surface under certain conditions.^37^ In order to investigate whether the Nbs had an effect on GPVI shedding, which could cause loss of clusters, GPVI surface expression was measured using flow cytometry after pre‐incubation of platelets with the Nbs. NEM, a thiol‐modifying compound, which activates metalloproteinases and causes strong shedding of GPVI,^38^ was used as a positive control. Figures 6D and 6E show no effect of either Nb2 or Nb53 on GPVI surface expression, whereas NEM caused complete loss of the receptor. These results show that Nb2 did not cause receptor shedding but effectively disrupted GPVI clustering along collagen fibers as well as prevented thrombus formation, demonstrating that clustering is important for thrombus formation under flow.

## 4 DISCUSSION

In the present study we used atherosclerotic plaque material as a physiologically relevant substrate to demonstrate that anti‐GPVI Nbs can be used as effective inhibitors and imaging tools to study GPVI localization and function. Nb2 potently inhibited GPVI‐mediated thrombus formation, disrupted GPVI clustering along collagen I fibers, and blocked GPVI downstream signaling in response to collagen I and plaque homogenate but did not induce receptor shedding. In addition, we showed non‐inhibitory Nb28 can be used to provide mechanistic insight by visualizing GPVI localization in platelets in whole blood under flow, without interfering with the inhibitory activity of Nb2 on GPVI clustering. Furthermore, we demonstrate atherosclerotic plaque preparations exhibit strong thrombogenic potential, which is mediated through GPVI and is independent of individual integrins. Taken together the present work underlines the central role of GPVI in platelet activation via atherosclerotic plaque and supports the further development of Nb2 as a promising antithrombotic agent.

Human atherosclerotic plaque has previously been utilized as a physiological platelet agonist; however, there is evidence for heterogeneity.^3^, ^7^, ^11^, ^39^ In order to limit this, we formed a pooled homogenate of 10 human atherosclerotic plaque samples. Collagen has been demonstrated to be the main platelet agonist present in plaque material, with evidence showing modification of plaque collagens results in their specificity for GPVI.^3^, ^7^, ^8^, ^9^, ^11^ GPVI blockade or depletion in human and murine platelets has been shown to result in almost complete inhibition of plaque induced‐thrombus formation, while integrin α2β1 inhibition had no effect.^7^, ^8^, ^11^ Nb2 was able to block GPVI downstream signaling to both collagen I and plaque, and effectively reduced total platelet tyrosine phosphorylation to plaque, but not to collagen I, reflecting the activation of other receptors by this ligand. We have previously shown that Nb2 binds GPVI at a site adjacent to, but not overlapping with, the synthetic collagen related peptide (CRP) binding site and this causes a slight conformational change.^25^ Nb2 can also outcompete GPVI for collagen I binding in a solid‐phase binding assay.^25^ Addition of Nb2 to already spread platelets did not have a large effect, only reducing the percentage of Ca^2+^ spiking platelets on collagen I, but not on plaque. As collagen has been proposed to be altered in plaque,^3^, ^7^, ^9^ this could indicate that GPVI binds more tightly and cannot therefore be displaced by the addition of Nb2. Under shear, Nb2 abolished thrombus formation on both plaque and collagen I, yet adhesion was only affected on plaque. Platelet activation markers, particularly the highly GPVI‐dependent PS exposure,^40^, ^41^, ^42^ were also markedly decreased on both surfaces with Nb2 treatment. This is similar to that seen with the anti‐GPVI F(ab)' fragment of mAb 9O12,^8^ the precursor antibody to the humanized form ACT017 (Glenzocimab).^43^ Combined inhibition of the two main platelet integrins α2β1 and αIIbβ3 was required to produce similarly strong inhibition of thrombus formation and platelet activation, but lacked the effect on procoagulant platelet formation achieved by Nb2. As eptifibatide treatment causes excessive bleeding,^44^ combined inhibition of αIIbβ3 and α2β1 would not be a viable treatment strategy to prevent thrombus formation. Inhibiting individual integrins did not result in any major effects on platelet adhesion and thrombus formation on plaque under flow. This lack of effect of integrin inhibition, together with the potent inhibition by Nb2, further illustrates the GPVI dependence of platelet activation by plaque.

The small size, stability, and high affinity of Nbs make them ideal reagents for imaging of receptors.^27^, ^29^ Receptor clustering is important to sustain and amplify signaling in many different cell types, for example antigen receptors in lymphocytes,^45^ and there are indications this is also the case for GPVI.^18^, ^19^, ^20^ However, the GPVI studies were based on static spreading of washed platelets on collagen. Here we have shown for the first time, using fluorescently labeled Nb28, that GPVI clusters along exposed collagen I fibers in whole blood under shear. In addition, using labeled Nb28 we also showed Nb2 disrupted this GPVI clustering. Inhibition of integrin α2β1 under flow did not affect GPVI clustering or multilayer thrombus formation on collagen, although thrombus number and activation markers were still reduced, showing the combined activation of both receptors is required for full thrombus formation on collagen.^14^ We have previously shown that platelets spread on fibrillar collagen I, exhibited clustered GPVI, and sustained GPVI‐dependent signaling.^19^, ^20^ We now show this clustering and sustained signaling is important for the build‐up of platelet aggregates and PS exposure on collagen under flow, as Nb2 treatment does not affect platelet adhesion to collagen I, but abolishes multilayered thrombus formation and PS exposure.

More targeted approaches to manage atherothrombosis are needed as current treatments cause increased bleeding.^46^ GPVI is a target for new therapies due to its platelet specificity,^12^ major role in plaque activation,^7^, ^8^, ^10^, ^11^ and minimal involvement in hemostasis.^16^, ^22^, ^23^, ^40^ The anti‐GPVI F(ab) Glenzocimab (formerly ACT017) is already undergoing clinical trials for treatment of acute ischemic stroke.^47^, ^48^ Nb2 has a high binding affinity for GPVI, with an equilibrium dissociation constant (*K*_D_) of 0.7 nM,^25^ compared to ACT017 (K_D_ <8 nM).^49^ Nbs have been shown to have a relatively short half‐life when used *in vivo* as they are quickly cleared from the system through the kidneys due to their small size. This can be counteracted by linkage to a larger inert molecule, such as serum albumin, or by making multimeric forms of the Nb.^50^ Yet, their short half‐life could provide a safety benefit in the acute setting of acute coronary syndromes or stroke, as any possible adverse effects on hemostasis would wear off more quickly in the event of bleeding. Several Nbs have already made the transition into the clinic by entering clinical trials.^50^ The success of the bivalent Nb against von Willebrand factor, caplacizumab, which is used to treat the rare genetic disease, immune thrombotic thrombocytopenic purpura^51^, ^52^ sets a good precedent for the use of Nbs to treat platelet‐related disorders. Nbs are also very stable and resistant to extremes in pH and temperature and are therefore more amenable to different drug delivery methods. Indeed, caplacizumab is delivered both intravenously and subcutaneously.^50^, ^52^ In addition, the relative ease of Nb production in microbial expression systems has cost benefits in the large‐scale production required for therapeutic uses.^53^

In summary, we demonstrate anti‐GPVI Nbs are excellent new research tools that can be used to visualize or inhibit platelet GPVI. Nb2 effectively blocked GPVI‐mediated platelet activation and thrombus formation by collagen and atherosclerotic plaque while fluorescent labelling of non‐inhibitory Nb28 enabled visualization and interrogation of GPVI clustering in more physiological conditions of whole blood under shear. The potent inhibition of atherosclerotic plaque‐induced thrombus formation by Nb2 advocates for its further investigation as a potential anti‐thrombotic treatment.

## AUTHOR CONTRIBUTIONS

N.J. Jooss designed and performed experiments, analyzed data, prepared figures, and wrote the manuscript. C.W. Smith performed experiments and wrote the manuscript. A. Slater and S.J. Montague performed experiments and revised the manuscript. Y. Di generated nanobodies. C. O'Shea wrote the MATLAB code for Ca^2+^ mobilization analysis. M.R. Thomas provided plaque material, and revised the manuscript. Y.M.C. Henskens, J.W.M. Heemskerk, and S.P. Watson designed experiments, provided funding and supervision, and revised the manuscript. N.S. Poulter designed and performed experiments, analyzed data, provided supervision and funding, and wrote the manuscript. All authors have read and approved the manuscript.

## CONFLICTS OF INTEREST

AS, MRT, SPW, and NSP have a patent for the anti‐GPVI nanobodies: WO2022/136457. The other authors have no conflicts of interest to declare.

## Supporting information


Appendix S1

Video S1

## Data Availability

For original data, please contact corresponding author.
