# Supplementary material for: Anti‐GPVI nanobody blocks collagen‐ and atherosclerotic plaque–induced GPVI clustering, signaling, and thrombus formation
Source: J Thromb Haemost. 2022 Aug 12;20(11):2617–31. doi: 10.1111/jth.15836 (PMC9804350; doi:10.1111/jth.15836)
Supplement: Supplementary file 1 — Appendix S1 [file JTH-20-2617-s001.docx]

**Supplementary Materials**

**Anti-GPVI nanobody blocks collagen- and atherosclerotic plaque-induced GPVI clustering, signaling and thrombus formation**

Natalie J. Jooss,^1,2^ Christopher W. Smith, ^1^ Alexandre Slater, ^1^ Samantha J. Montague, ^1^ Ying Di, ^1^ Christopher O’Shea, ^1^ Mark R. Thomas,^1, 3^ Yvonne Henskens,^4^ Johan W. M. Heemskerk,^2,5^ Steve P. Watson,^1,6^ Natalie S. Poulter^1,6^

^1^Institute of Cardiovascular Sciences, College of Medical and Dental Sciences, University of Birmingham, Edgbaston, Birmingham, UK. ^2^Department of Biochemistry, Cardiovascular Research Institute Maastricht (CARIM), Maastricht University, Maastricht, The Netherlands. ^3^ Department of Cardiology, University Hospitals Birmingham, Birmingham. ^4^Central Diagnostic Laboratory, Maastricht University Medical Centre, Maastricht, the Netherlands. ^5^Synapse Research Institute Maastricht, Kon. Emmaplein 7, 6214 AC Maastricht, the Netherlands.^6^Centre of Membrane Proteins and Receptors (COMPARE), Universities of Birmingham and Nottingham, Midlands, UK.

**Corresponding author:**

Natalie S. Poulter,

**Address**: ^1^Institute of Cardiovascular Sciences, College of Medical and Dental Sciences, University of Birmingham, Edgbaston, Birmingham B15 2TT, West Midlands, UK

**Email**: [n.poulter@bham.ac.uk](mailto:n.poulter@bham.ac.uk)

**Tel:** 0121 415 8678

**Supplemental Methods**

Platelet spreading and imaging

Glass coverslips were coated with 10 µg/ml Fibrillar Collagen I or 500 µg/ml pooled plaque homogenate at 4°C overnight, then blocked with 5 mg/ml heat-denatured bovine serum albumin (BSA) for 60 minutes. Washed platelets (2x10^7^/ml) were allowed to spread for 45 minutes at 37°C, after preincubation with either PBS or 500 nM Nb2 for 10 minutes. Next, cells were fixed with 5% formalin (10 minutes), permeabilized with 0.1% Triton (5 minutes) and labeled for 1 hour at RT with phalloidin-Alexa488 in PBS with 3x PBS washes between each step. Coverslips were mounted onto microscope slides using Hydromount (National Diagnostics). Samples where imaged using a 63x 1.4NA oil immersion objective lens on a Zeiss Axio Observer 7 Epifluorescence microscope equipped with a Colibri 7 LED light source, Zeiss filter sets 38 for GFP/FITC and a Hamamatsu ORCA Flash 4 LT sCMOS camera for image acquisition. Image processing was done in FIJI 1.52a (NIH, Bethesda, USA). Platelet spreading analysis was carried out on all the platelets in 6 fields of view, per condition per experiment, as described in Pike *et al.*^3^ .

Ca^2+^ mobilization in live spread platelets

Glass bottom dishes (MatTek Corp) were coated and blocked, as above. Freshly prepared washed platelets were loaded with 1 μM Oregon Green-488 BAPTA-1-AM (ThermoFisher) at 37°C for
45 minutes. Excess dye was removed by addition of 25 ml modified Tyrode’s-HEPES buffer, 3ml ACD and 2.8 μM prostacyclin, followed by 10 minutes of centrifugation at 1000 x g, and resuspension in modified Tyrode’s-HEPES buffer. Dye-loaded platelets (2x10^7^/ml) were allowed to spread in MatTek dishes for 45 minutes at 37°C. Two videos (1 frame per second for 2 minutes) were acquired both before and after addition of either 500 nM Nb2 or Nb53 using a Zeiss Axio Observer 7 epifluorescence microscope and 63 × 1.4 N.A. objective and GFP filter set, as detailed above. Videos of Ca^2+^ mobilization were analyzed by manually drawing of ~30 platelets per field of view, and generation of fluorescence intensity profiles for each platelet in ImageJ v1.52a. If there were more than 30 platelets in a FOV a grid pattern was applied to the image and platelets in the top left-hand corner of the image were selected first, with the area included increasing by one grid square to the right and below until 30 platelets were selected. The percentage of spiking platelets, as well as average duration and amplitude of the spikes were quantified using a MATLAB code adapted from Pallini *et al.* ^4^, where a change in intensity was identified as a Ca^2+^ spike if it increased by greater than 25% above baseline fluorescence intensity for each individual cell. A spike was defined to have ended once fluorescence intensity had decreased by 80% of spike amplitude. Data were exported to Microsoft Excel for further analysis.

Western blotting

Washed platelets (5x10^8^/ml) were preincubated with either PBS or 500 nM Nb2 or Nb53 for
10 minutes, before being stimulated with 10 µg/ml Fibrillar Collagen I or 500 µg/ml pooled plaque homogenate at 1200 rpm and 37°C for 180 seconds on a shaking plate incubator (Eppendorf) in the presence of 9µM Eptifibatide. Whole cell lysates were generated by addition of 5X SDS reducing sample buffer. Lysates were subjected to sodium dodecylsulfate polyacrylamide gel electrophoresis (SDS-PAGE) and western blotting. PVDF membranes were blocked (5% BSA in TBST) and then incubated at 4°C overnight with antibodies (diluted in block) against phospho-tyrosine (1:1000, 4G10, Millipore, 05-321), phospho-PLCγ2 (1:250, Y1217, Cell Signalling, 3871S), phospho-LAT (1:500, Y200, Abcam, ab68139), phospho-Syk (1:500, Y525/526, Cell Signalling, 2710S) as well as total Syk (1:500, 4D10, St. Cruz, sc1240) or total LAT (1:500, Merck-Millipore, 06-807) as a loading control. Membranes were washed and incubated with HRP labelled secondary antibody at room temperature for 1 hour, and developed using SuperSignal™ West Pico PLUS Chemiluminescent Substrate (Thermo Scientific Pierce). Results were visualized on film, as well as imaged for quantification with an Odyssey Fc System (LI-COR Biosciences) in combination with Image studio lite v5.2.

ELISA competition assay

The binding of 100 nM Nb28 and Nb21 to immobilized GPVI-Fc (10 nM) in the presence of increasing concentrations of non-tagged Nb2 (0-2000 nM) was assessed using a competitive surface binding assay as previously described ^5^. Binding of Nb28 and 21 was detected using HRP conjugated anti-His secondary antibody.

Whole blood microfluidics

Whole blood samples (500 µl) were perfused over 2 microspots through a Maastricht parallel flow chamber at a shear rate of 1000/s at room temperature, as described elsewhere ^6^. In brief, degreased glass coverslips were coated with 0.5µl microspots of 100 µg/ml fibrillar collagen I and 500 µg/ml pooled plaque homogenate overnight; and then blocked with 1% BSA in HEPES buffer (10 mM HEPES, 136 mM NaCl, 2.7 mM KCl, 2 mM MgCl_2,_ pH 7.45) for 30 minutes. Citrated whole blood samples were thrombin inhibited (40 µM PPACK) and recalcified (3.75 mM MgCl_2_ and 7.5 mM CaCl_2_). The blood was preincubated for 10 minutes with either vehicle or 500 nM Nb2 or Nb53, prior to perfusion through the flow chamber for 3.5 minutes. Two endpoint brightfield images were taken, while flowing labeling buffer: HEPES buffer, 2 mM CaCl_2_, 1 unit/ml heparin, 5.5 mM glucose, 0.1% BSA and AF568-annexin A5 (for phosphatidylserine exposure, ThermoFisher), AF647 anti-CD62P mAb (for CD62P expression, BioLegend), and anti-fibrinogen FITC Ab (for integrin αIIbβ3 activation, DAKO) for 1.5 min. Unbound label was washed off, for 2 min, with rinse buffer (HEPES buffer, 2 mM CaCl_2_, 1 unit/ml heparin, 5.5 mM glucose and 0.1% BSA), and endpoint fluorescence images of three random fields of view were acquired with an EVOS AMF4300 microscope (Life Technologies). Brightfield images were quantified with two semi-automated ImageJ scripts generated in-house, to assess the percentage surface area covered by platelets (platelet deposition) as well as the surface area covered by thrombi (multilayered thrombi). For expression of the platelet activation markers (integrin activation, P-selectin expression and PS exposure) three more in-house generated semi-automated ImageJ scripts were employed to assess the percentage of the surface area covered by the fluorescent marker^7^. All raw values are averaged between images as well as runs, per donor. Next subtraction heatmaps were made using the program R. Average raw values over all donors and the two substrates, were univariate-normalized at a scale of 0–10 for each parameter. Control values were then subtracted from the treatment values, and differences that were statistically significant (P<0.05) by a one-way ANOVA were then summarized in the heatmaps to only visualize relevant effects. Green represents inhibitory effects and red represents activation.

References

**1.** Nicolson PLR, Hughes CE, Watson S, et al. Inhibition of Btk by Btk-specific concentrations of ibrutinib and acalabrutinib delays but does not block platelet aggregation mediated by glycoprotein VI. *Haematologica.* Dec 2018;103(12):2097-2108.

**2.** Nicolson PLR, Nock SH, Hinds J, et al. Low-dose Btk inhibitors selectively block platelet activation by CLEC-2. *Haematologica.* Jan 1 2021;106(1):208-219.

**3.** Pike JA, Simms VA, Smith CW, et al. An adaptable analysis workflow for characterization of platelet spreading and morphology. *Platelets.* Jan 2 2021;32(1):54-58.

**4.** Pallini C, Pike JA, O'Shea C, et al. Immobilized collagen prevents shedding and induces sustained GPVI clustering and signaling in platelets. *Platelets.* Jan 2 2021;32(1):59-73.

**5.** Slater A, Di Y, Clark JC, et al. Structural characterization of a novel GPVI-nanobody complex reveals a biologically active domain-swapped GPVI dimer. *Blood.* Jun 17 2021;137(24):3443-3453.

**6.** de Witt SM, Swieringa F, Cavill R, et al. Identification of platelet function defects by multi-parameter assessment of thrombus formation. *Nat Commun.* Jul 16 2014;5:4257.

**7.** van Geffen JP, Brouns SLN, Batista J, et al. High-throughput elucidation of thrombus formation reveals sources of platelet function variability. *Haematologica.* Jun 2019;104(6):1256-1267.

**Supplemental Figures**

**
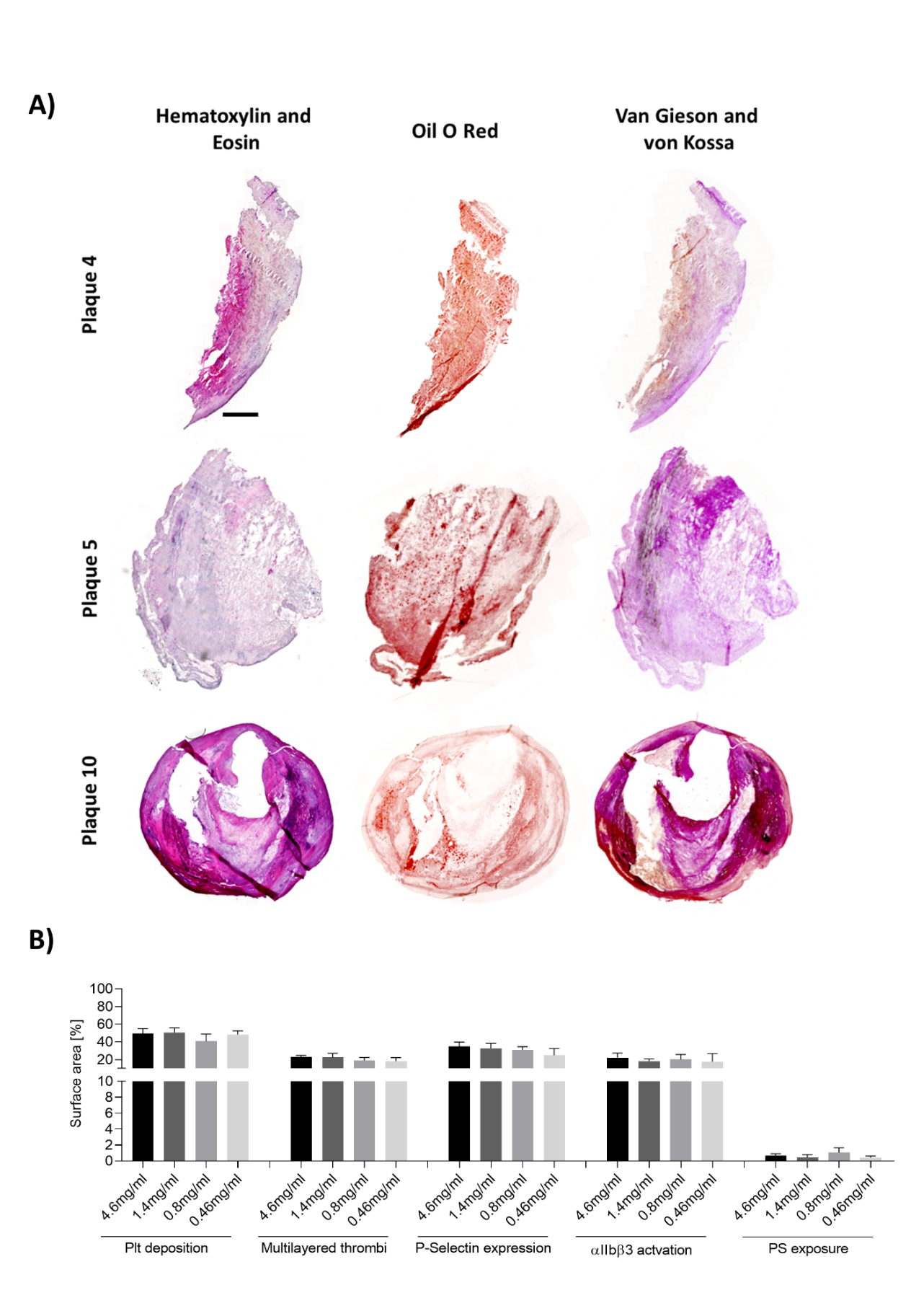
**

**Supplementary Figure 1: Atherosclerotic plaques exhibit heterogeneity in composition and plaque homogenate displayed no dose dependent thrombus formation and platelet activation under flow at tested concentrations. A)** Half of all human plaques present in the pooled plaque homogenate were OCT imbedded, cryosectioned at 6µm and following stained to assess general orientation of the plaque with Hematoxylin and Eosin for nuclear and cellular compounds; with OilORed to visualize lipid deposition; as well as a double stained with Van Gieson and von Kossa for global collagen content (in pink) and Ca^2+^ deposition (in black), respectively. Displayed here are all stains of 3 representative plaques showing different distribution and overall presence of corresponding markers. Scale bar = 500µM. **B)** Based on literature, 4 different dilutions of pooled plaque homogenate were coated on glass coverslips and whole blood was perfused through parallel flow chamber at 1000/s. Thrombus parameters as well as expression of platelet activation markers were assessed. n=1. SAC= Surface Area Coverage.

**
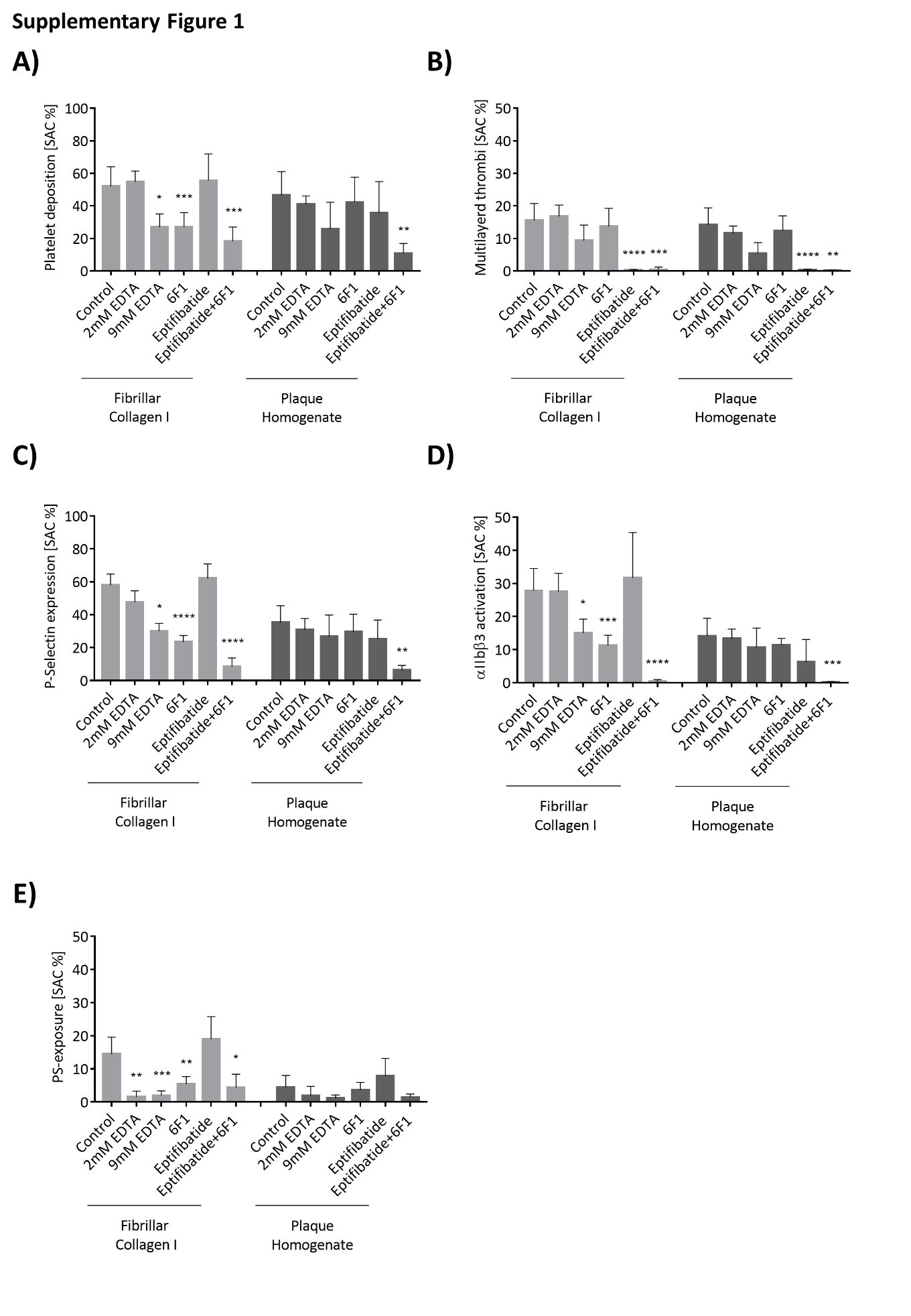
**

**Supplementary Figure 2: Platelet activation induced by plaque homogenate is independent of individual integrin inhibition.** Whole blood was perfused over fibrillar collagen I as well as plaque homogenate after pre-incubation for 10 minutes with 2mM or 9mM EDTA, 20 µg/ml 6F1, 9 µM Eptifibatide or 20 µg/ml 6F1 and 9 µM Eptifibatide. Surface area covered by platelets (**A**) or multilayer thrombi (**B**) was extracted from brightfield images. Fluorescence images were quantified for P-Selectin expression (**C**), αIIbβ3 activation (**D**) and PS-exposure (**E**) with semi-automated ImageJ scripts. SAC= Surface Area Coverage. n=3-5. One-way ANOVA. *P<0.05, **P<0.005, ***P<0.0005, ****P<0.0001.


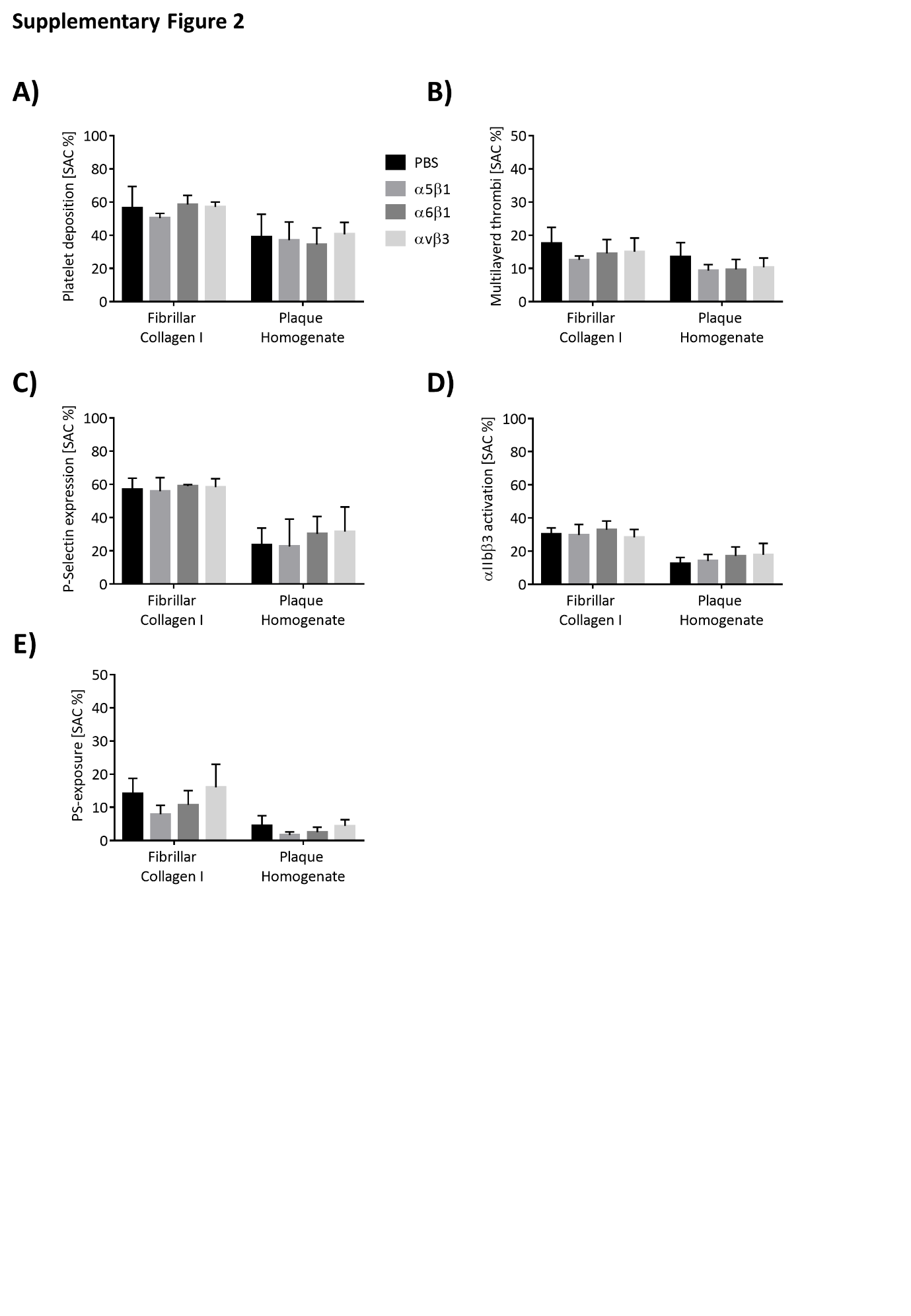


**Supplementary Figure 3: Inhibiting integrins α5β1, α6β1 and αvβ3 individually does not affect thrombus formation**. Whole blood was perfused over fibrillar collagen I as well as plaque homogenate after pre-incubation for 10 minutes with 20 µg/ml α5β1 or α6β1 or 100 µM αvβ3 inhibitors. Surface area covered by platelets (**A**) or multilayer thrombi (**B**) was extracted from brightfield images. Fluorescence images were quantified for P-Selectin expression (**C**), αIIbβ3 activation (**D**) and PS-exposure (**E**) with semi-automated ImageJ scripts. n=3-5. SAC= Surface Area Coverage. One-way ANOVA, all changes shown were non-significant.

**
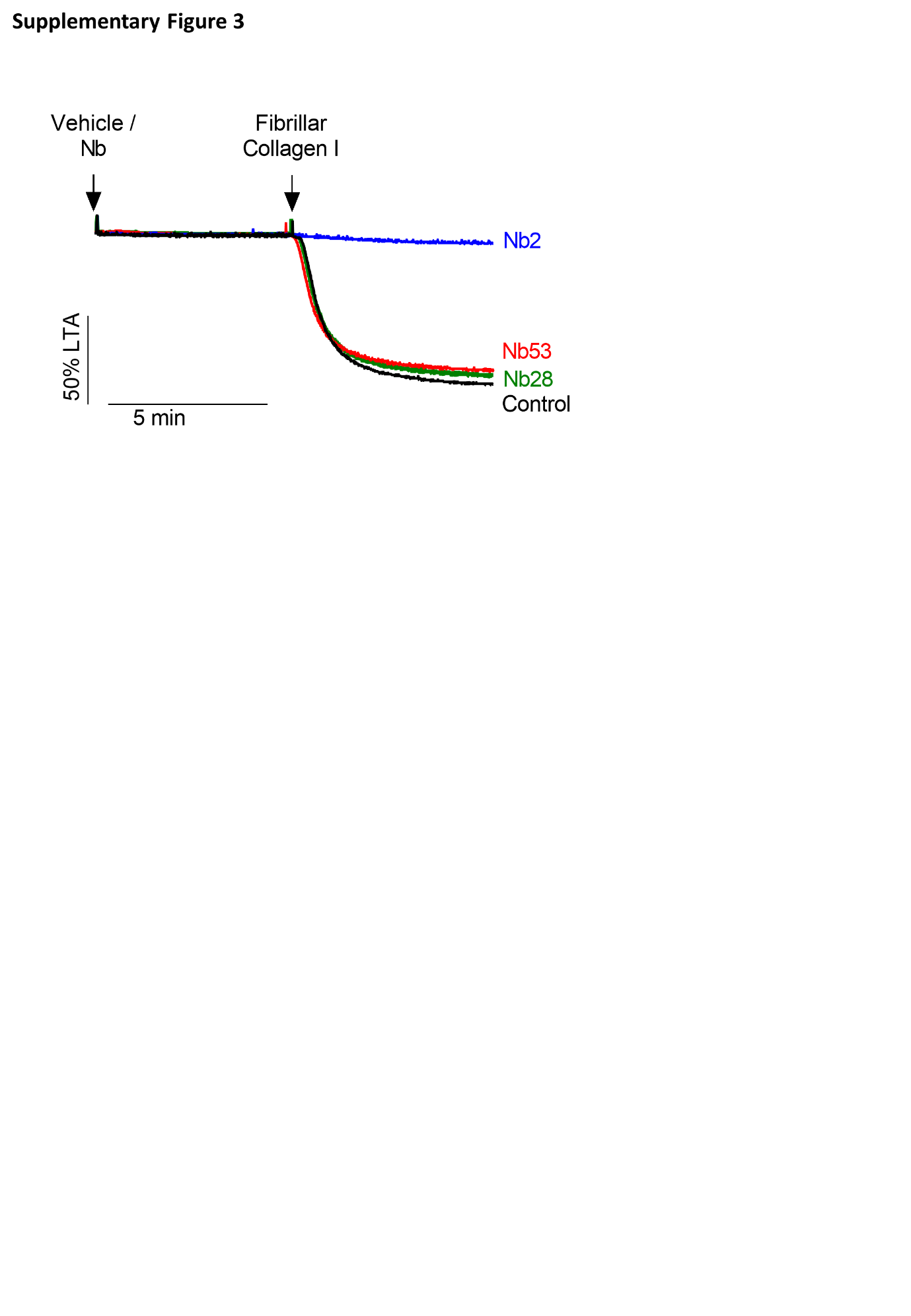
**

**Supplementary Figure 4: Nb28 and Nb53 do not inhibit collagen-induced platelet aggregation.** Washed platelets were preincubated with 500nM of either Nb2, Nb28 or Nb53 for 6 mins prior to addition of 10μg/ml fibrillar collagen I and platelet aggregation measured by light transmission aggregometry for 10 min.


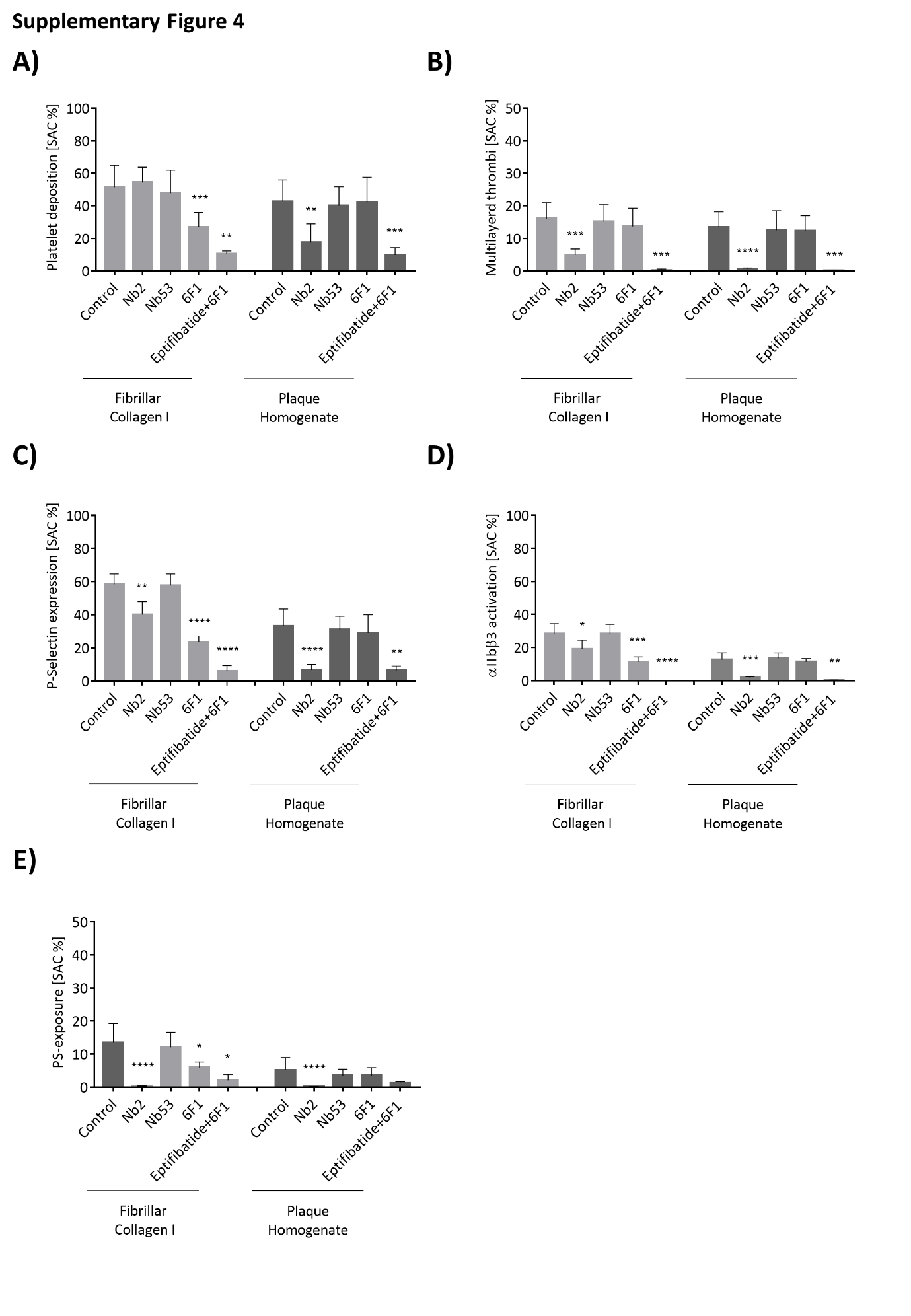


**Supplementary Figure 5: Anti-GPVI Nb2 inhibits platelet activation at arterial shear rates.** Whole blood was perfused over Fibrillar Collagen I as well as Plaque Homogenate after pre-incubation for 10 minutes with 500 nM Nb2 or negative control Nb53, 20 µg/ml 6F1 or 20 µg/ml 6F1 and 9 µM Eptifibatide. Surface area covered by platelets **(A)** or multilayer thrombi **(B)** was extracted from brightfield images. Fluorescence images were quantified for P-Selectin expression **(C)**, αIIbβ3 activation **(D)** and PS-exposure **(E)** with semi-automated ImageJ scripts. n=3-5. SAC= Surface Area Coverage. One-way ANOVA. *P<0.05, **P<0.005, ***P<0.0005, ****P<0.0001.

**Supplemental Video 1: Plaque homogenate induces Ca^2+^ mobilization in spread platelets.** Representative videos of Ca^2+^ spiking in platelets loaded with 1 μM BAPTA-Oregon green Ca^2+^ indicator dye and spread on plaque homogenate (500 µg/ml) (left) and fibrillar collagen I (10 μg/ml) (right) for 45 minutes prior to imaging. Images were taken every second for 2 min (n=7). Scale bar= 20 μm.
